# Hyperbaric oxygen treatment of spinal cord injury in rat model

**DOI:** 10.1186/s12883-017-0909-z

**Published:** 2017-07-03

**Authors:** Yongming Sun, Dong Liu, Qingpeng Wang, Peng Su, Qifeng Tang

**Affiliations:** 1 Department of Orthopaedic Surgery, The Second Affiliated Hospital of Soochow University, Suzhou, Jiangsu 215006 China; 2 Department of Anesthesiology, Suzhou BenQ Medical Center, Nanjing Medical University, Suzhou, 215009 China

**Keywords:** Hyperbaric oxygen, Spinal cord injury, Oxygen free radicals

## Abstract

**Background:**

The purpose of this study was to investigate the therapeutic effects and mechanisms of hyperbaric oxygen (HBO) treatment on rats following spinal cord injury (SCI).

**Methods:**

A total of 45 Sprague-Dawley (SD) rats were randomly divided into three groups. Sham-SCI group was surgically exposed but not subjected to the SCI procedure. SCI-control group was administered SCI and treated with regular air. SCI-HBO group was administered SCI and HBO treatment. Neuromotor functions were examined using the Basso, Beattie, and Bresnahan (BBB) locomotor rating scale and the inclined plane assessment at before SCI (baseline) and after SCI. Superoxide dismutase (SOD) activities and malondialdehyde (MDA) levels were measured.

**Results:**

Starting from Day 1 after SCI but except Day 2, the SCI-HBO group has significantly higher BBB scores than the SCI-control group. After SCI, the maximum inclination angles at which rats could maintain were significantly lower in both SCI groups. But the maximum angles were significantly bigger for the rats in the SCI-HBO group than those on the SCI-control group at 5, 10 and 20 days after SCI. SOD activities in SCI-HBO rats were significantly higher and MDA levels were significantly lower than in SCI-control rats, at two and five days after SCI. There was also less cystic degeneration of spinal cord in SCI-HBO rats, compared to SCI-control rats.

**Conclusions:**

These results suggest that HBO treatment has a therapeutic value in treating SCI. Increased oxygen free radical scavenging and reduced lipid oxidation may be one of the mechanisms.

## Background

Spinal cord injury (SCI) often leads to paralysis and high morbidity. After SCI, a series of pathophysiological responses lead to progressive spinal cord tissue degeneration and necrosis, likely due to microcirculation disorders and neuron biochemical imbalance involving prostaglandins, calcium, neurotransmitters, and free radicals, considered one of the most important factors causing spinal cord tissue necrosis and degeneration [1]. Neuronal cell membrane structures are rich in lipids, and research has found that lipid oxidation caused by free radicals has important implications on SCI outcome [1, 2]. While some studies into SCI treatment have found that hyperbaric oxygen (HBO) can reduce the generation of oxygen free radicals in the body, thus reducing the oxygen free radicals caused lipid oxidation and accelerating SCI repair, other studies found opposite results [3–5]. Therefore, HBO effect on nerve injury is yet to be elucidated.

This study investigates the HBO treatment of SCI in an experimental rat SCI model and examines HBO effect on the recovery of neuromotor functions. Serum malondialdehyde (MDA) and superoxide dismutase (SOD) levels were also measured. MDA is a lipid oxidation product generated through lipid peroxidation. The spinal cord neurons contain membrane structures rich in lipid, the oxidation of which during SCI further exacerbates neuronal damage. SOD is an enzyme that participates in superoxide radical scavenges that maintains the cellular oxidative and anti-oxidative balance, thus helping to eliminate the free radical damage to the cells and maintain normal cell functions. Therefore, the SOD and MDA levels in the body are a reflection of the extent of lipid peroxidation in injured cells [2]. The joint determination of SOD activity and MDA content may indirectly reflect the extent of the free radical damage of the neuronal cells caused by SCI. Our hypothesis is that HBO treatment reduces lipid oxidation induced by SCI, thus accelerating recovery of neuromotor functions after SCI.

## Methods

### Experimental animals

The Ethics Committee of the Second Affiliated Hospital of Soochow University approved the protocol of the present study. A total of 45 healthy Sprague-Dawley female rats of clean-grade (15 rats in each group), 3 to 4 months old and weighing 215 to 300 g, were provided by the Experimental Animal Center of Suzhou University. During the experiments, the animals were treated in compliance with the “Guiding Opinions on the Ethical Treatments of Laboratory Animals” published by the Ministry of Science and Technology in 2006. All rats were randomly divided into three groups. Sham-SCI group was surgically exposed, but not subjected to the SCI procedure. SCI-control group was administered SCI and treated with regular air. SCI-HBO group was administered SCI and HBO treatment.

### Experimental spinal cord injury model

In SCI-control group and SCI-HBO group, rats were administered SCI using the modified Allen weight-drop method [6]. Briefly, rats were injected intraperitoneally with 3.6% chloral hydrate (1 ml / 100 g) under general anesthesia, and the spinal cord T11–12 plane area (about 4 mm × 8 mm) were exposed. A 2.5 mm diameter metal cylinder, weighing 10 g, was dropped from a height of 6.5 cm in a vertical plastic tube, directly onto the exposed spinal cord, resulting in acute moderate SCI with an injury force of 6.5 g*cm.

### Neuromotor function assessment

The neuromotor functions of all the rats were examined by two technicians not involved in this study, using the Basso, Beattie, and Bresnahan (BBB) locomotor rating scale and the inclined plane assessment, at baseline, 1 h, 2, 5, 10 and 20 days after SCI [7, 8]. For the inclined plane assessment, the inclination angle of the board is freely adjustable. The maximum inclination angle of the board on which the rat can stay for 5 s without falling off was recorded. The BBB rating scale has a range from zero to 21 points, judged by parameters such as coordination of limb movement, paw placement and tail balance. No visible movement of the hind legs is scored as 0 points. For the maximum 21 points, the rat has to walk continuously on the paws, with a cocked tail, good fore and hind limb motor coordination and truck stability. The toes have to grip the surface while moving forward and the paws maintain coordination with the body movement.

### Hyperbaric oxygen (HBO) treatment

In the SCI-HBO group, HBO treatment began at two hours after SCI. A single-person medical hyperbaric oxygen chamber (Ningbo hyperbaric oxygen corporation, China) was prepared with flush of pure oxygen for ten minutes. The rats in the SCI-HBO group was put into the HBO chamber and exposed to 80% oxygen at 0.3 MPa (3ATA) for 60 min, followed by depress urization for 30 min. The HBO therapy was carried out once a day, for five days. The rats of SCI-control group treated with regular air.

### Superoxide dismutase (SOD) and malondialdehyde (MDA) assay and spinal cord pathology

Tail blood (1.5 ml) was taken at baseline, two and five days after SCI, from which SOD activities and MDA levels were measured using the xanthine oxidase method and the thiobarbituric acid method (Nanjing Jiancheng Bioengineering Institute, China), respectively. At 20 days after SCI, the rats were sacrificed and the SCI pathology was examined with HE staining. Proteins were prepared from spinal cord tissue obtained from the lesion epicenter (2.5 mm cephalad and caudally).

### Statistical analysis

All data are presented as mean ± standard deviation (SD). Statistical analyses were performed with the Prism software package (GraphPad v5, San Diego, CA, USA). Data were analyzed using two-way repeated measures ANOVA with bonferroni post-hoc testing, and then the Newman-Keuls test for multiple comparisons. A *P*-value less than 0.05 were accepted as statistically significant.

## Results

### Hind limb neuromotor function assessment

**Fig. 1:**
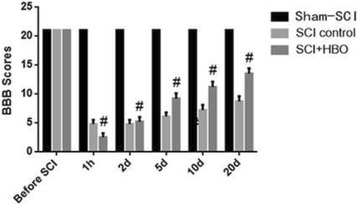
The BBB Scores of animals pre- and post SCI surgery. (compared to the control group, # *P* < 0.05)

Rats before SCI showed the baseline results. Rats in both the SCI-HBO group and the SCI-control group had BBB scores of 21 points at baseline. At one hour after SCI, the BBB scores were dramatically lowered to 1.7 ± 0.6 in the SCI-control group and 2.5 ± 0.7 in the SCI-HBO group, respectively. There is no significant difference in the BBB scores between the HBO and control groups. Starting from Day 1 after SCI but except Day 2, the SCI-HBO group has significantly higher BBB scores than the SCI-control group. At Day 20 after SCI, the BBB scores were 13.5 ± 0.9 in the SCI-HBO group and 8.7 ± 0.9 in the SCI-control group, respectively. Sham-SCI rats had BBB scores of 21 throughout the study (Fig. 1) (Tables 1 and 2).

**Table 1:** BBB scores of sham-SCI, SCI-control and SCI-hyperbaric oxygen (HBO) rats

Grouping	*n*	Before SCI	1 h	2 d	5 d	10 d	20 d
sham-SCI	15	21.0 ± 0	21.0 ± 0	21.0 ± 0	21.0 ± 0	21.0 ± 0	21.0 ± 0
SCI-control	15	21.0 ± 0	1.7 ± 0.6	4.8 ± 0.7	6.1 ± 0.7	7.2 ± 0.9	8.7 ± 0.9
SCI-HBO	15	21.0 ± 0	2.5 ± 0.7^#^	5.2 ± 0.8	9.2 ± 0.9^#^	11.2 ± 0.9^#^	13.5 ± 0.9^#^

Mean values ± SD

Compared to the control group, #*P* < 0.05

**Table 2:** Two-way repeated measures ANOVA

Source	SS	v(df)	MS	*F*	*P*
Interaction	2288	10	228.8	*F* (10, 252) = 631.7	*P* < 0.0001
Row Factor	8370	2	4185	*F* (2, 252) = 11,553	*P* < 0.0001
Column Factor	4264	5	852.7	*F* (5, 252) = 2354	*P* < 0.0001
Residual	91.28	252	0.3622		

BBB scores of different treatment methods have significant differences, HBO increased the BBB score after SCI and different time after treatment. The difference was significant; different treatment methods and time have interactive effects

**Fig. 2:**
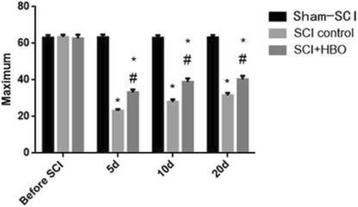
The inclination angle of animals pre- and post SCI surgery. (compared to the sham -SCI, * *P* < 0.05; compared to the control group, # *P* < 0.05)

For the inclined plane assessment, rats in both the SCI-HBO group and the SCI-control group achieved a maximum angle of 63 degrees at baseline. After SCI, the maximum inclination angles at which rats could maintain were significantly lower in both SCI groups. But the maximum angles were significantly bigger for the rats in the SCI-HBO group than those on the SCI-control group at 5, 10 and 20 days after SCI (Fig. 2) (Table 3).

**Table 3:** Maximum inclined plane angles of sham-SCI, SCI-control and SCI-HBO rats

Grouping	*n*	Before SCI	5 d	10 d	20 d
sham-SCI	15	63.1 ± 1.5	63.3 ± 1.5	63.0 ± 1.4	63.2 ± 1.3
SCI-control	15	63.4 ± 1.3	23.1 ± 0.9^*^	28.0 ± 1.3^*^	31.5 ± 1.4^*^
SCI-HBO	15	62.8 ± 1.9	33.2 ± 1.5^*#^	38.9 ± 1.9^*#^	40.3 ± 1.9^*#^

Mean values ± SD

Compared to the before SCI, *P < 0.05; compared to the control group, #*P* < 0.05

### Serum SOD and MDA content

**Fig. 3:**
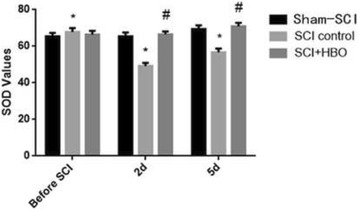
The SOD values of animals pre- and post SCI surgery. (compared to the sham -SCI, * *P* < 0.05; compared to the control group, # *P* < 0.05

**Fig. 4:**
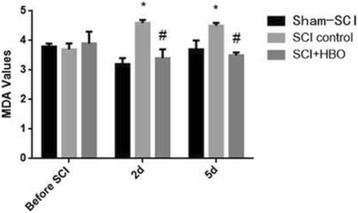
The MDA values of animals pre- and post SCI surgery. (compared to the sham -SCI, * *P* < 0.05; compared to the control group, # *P* < 0.05

xIn both groups of SCI rats, serum SOD levels were significantly decreased and serum MDA levels were significantly increased after SCI, compared to baseline. Serum SOD activities in the SCI-HBO rats were significantly higher than those in the SCI-control rat at two days after SCI (66.50 ± 1.72 vs 49.20 ± 1.69) and five days after SCI (70.90 ± 1.91 vs 56.70 ± 2.00). In contrast, MDA levels were significantly lower in the SCI-HBO rats than those in the SCI-control rats at 2 days after SCI (3.46 ± 0.37 vs 4.64 ± 0.12) and five days after SCI (3.58 ± 0.14 vs 4.55 ± 0.14). There was no change in both SOD and MDA levels in the sham-SCI rats (Figs. 3 and 4) (Table 4).

**Table 4:** SOD (unit/ml) and MDA (nmol/ml) values of sham-SCI, SCI-control and SCI-HBO rats

	Grouping	*n*	Before SCI	2 d	5 d
SOD	sham-SCI	15	65.4 ± 1.9	65.4 ± 2.1	69.4 ± 2.0
	SCI-control	15	67.8 ± 2.1	49.2 ± 1.7^*^	56.7 ± 2.0^*^
	SCI-HBO	15	66.4 ± 2.0	66.5 ± 1.72^#^	70.9 ± 1.9^#^
MDA	sham-SCI	15	3.8 ± 0.1	3.2 ± 0.2	3.7 ± 0.3
	SCI-control	15	3.7 ± 0.2	4.6 ± 0.1^*^	4.5 ± 0.1^*^
	SCI-HBO	15	3.9 ± 0.4	3.4 ± 0.3^#^	3.5 ± 0.1^#^

Mean values ± SD

Compared to the before SCI, *P < 0.05; compared to the control group, #*P* < 0.05

### Spinal cord histopathology

**Fig. 5:**
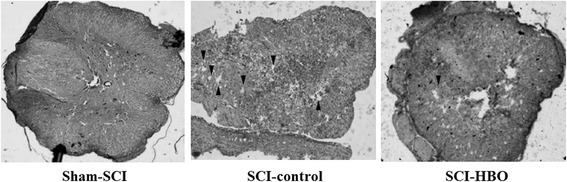
HE staining (×100) of spinal cord slices of rats in the sham-SCI, SCI-control and SCI-HBO groups at Day 20 after SCI, showing cystic degeneration (*arrow head*). Sham-SCI group: the nucleus of the spinal cord of normal rats was clear, and the *white* matter of the nerve fibers arranged in a dense and orderly fashion. SCI-control group: The *gray* matter in the spinal cord of SCI rats was disintegrated and necrotic, a great number of vacuoles formed. SCI-HBO group: The *gray* matter in the spinal cord of SCI rats was disintegrated and necrotic, a small number of vacuoles formed

Rats in both SCI groups had marked cystic degeneration, observed in the spinal cord HE staining, compared to the sham-SCI rats. The nucleus of the spinal cord of normal rats was clear, and the white matter of the nerve fibers arranged in a dense and orderly fashion. The gray matter in the spinal cord of SCI-control rats was disintegrated and necrotic, a great number of vacuoles were formed more than in HBO group. At 20 days after SCI, there was less cystic degeneration in the SCI-HBO rats than in the SCI-control rats (Fig. 5).

## Discussion

According to our clinical experience, acute SCI patients usually need at least 7 days for HBO treatment. For rats recover more quickly than men, the time should be shortened. Furthermore, HBO application of 5 days is similar to the clinical therapy. This is why we chose this therapy time window in our study.

In our study, SCI female rats in the HBO treatment group had significantly improved neuromotor function than the control non-HBO treated SCI rats at 5, 10 and 20 days after injury, judged by both higher BBB scores and larger inclination angles in the inclined plane assessment. Serum SOD activities were significantly higher and MDA levels were significantly lower in the SCI rats of the HBO group than those in the control group, at both 2 and 5 days post-SCI, suggesting an increase in SOD activities and reduced lipid peroxidation caused by oxygen free radicals. This was consistent with the accelerated recovery of neuromotor function in SCI rats with HBO treatment. Our results showed that there was a significant difference between the SCI-control and sham-SCI in baseline SOD serum levels indicating that this is likely to be a false positive result. The probable reason of this false result is the limited sample size. Although SOD levels of the SCI-control was higher than the sham-SCI and SCI was able to reduce serum SOD as previous studies, these results were in line with the effect of SCI on SOD.

Our previous study has demonstrated that cystic degeneration could be achieved by treatment of SCI [7]. And in this study, cystic degeneration was also observed from our results in the HBO treatment group. This histopathology changing indicated the curative effect of HBO.

MDA which is one of the most important products of membrane lipid peroxidation, exacerbates the damage of membrane. Therefore, it is necessary to study the mechanism of lipid peroxidation and the mechanism of lipid peroxidation. MDA plays an important role in mitochondrial respiratory chain complexes. The MDA content is a commonly used indicator in the study of plant senescence physiology and resistance physiology. SOD is a naturally occurring superoxide radical scavenger in the body, which can convert harmful superoxide radicals into hydrogen peroxide, and then protect the neuron cells. The combined determination of SOD activity and MDA can indirectly reflect the lipid peroxidation in vivo and the extent of the damage of the cells [2].

Many mechanisms may participate in the accelerated restoration of neuromotor function by HBO treatment of SCI rats: (1) increased supply of oxygen to mitigate hypoxia and edema in the damaged tissue; (2) inhibition of neuronal apoptosis through regulation of apoptosis-related genes, thus promoting recovery of the reversible neurological injury [9–14]; (3) increased expression of the hypoxia inducible factor to enhance hypoxia resistance [15]; (4) reversal of the ischemia-induced decline in the expression of neurotrophic factors, thus reducing the formation of scar tissue to promote recovery of neurological function [16]; (5) improvement of general antioxidant capacity of the body [4, 17]. Our study shows that in SCI rats with improved neuromotor function through HBO therapy, serum SOD activities increased and MDA levels decreased, indicating that the inhibition of oxygen free radicals may be one of the mechanisms by which HBO treatment improved neuromotor function in SCI rats. After SCI, large amounts of oxygen free radicals are generated and released in the spinal cord tissue, due to ischemia, anoxia, and neuron mitochondrial redox transport chain uncoupling [2]. The unstable unpaired electrons contained in the oxygen free radicals can attack the polyunsaturated fatty acids in the membrane structures of spinal cord neuronal cells, leading to lipid peroxidation, and cell and tissue damage [1]. The neuronal membrane structures in the spinal cord are rich in lipids, and the oxidation of which generates aldehydes, ketones, hydroxyls, carbonyls, hydrogen peroxide and new oxygen free radicals, further exacerbating cell damage [2, 18].

The effect of HBO treatment of acute SCI in clinical settings is controversial [3–5]. Our previous studies have shown that HBO treatment clinically accelerates the functional recovery in rats with SCI [19]. Acute SCI rats often have direct damage and even discontinuity of the spinal cord. The compression of spinal cord tissue by the fracture fragments results in local ischemia and local inflammatory response which led further spinal cord nerve tissue necrosis and apoptosis. So the most important clinical treatment of acute SCI is to repair the continuity of spinal cord, to relieve the compression of the local spinal cord tissue, and to further reduce the local and systemic inflammatory response. HBO therapy therefore is often not the immediate choice in acute SCI treatment. Nevertheless the neuronal tissue regeneration in SCI rats that require surgery remains a difficult task, due to the severe neuronal tissue damage; HBO therapy provides a better tissue environment for the repair and regeneration of injured spinal cord tissues, thus becoming part of a comprehensive effective treatment of SCI.

There are several weaknesses in our study. The modified Allen weight-drop method to inflict experimental SCI may cause only spinal shock or spinal cord concussion in some study rats, resulting in incomplete SCI. Secondly, the measurement of SOD and MDA were done at only Days 2 and 5 after SCI, compared to the neuromotor function assessment at up of Days 10 and 20 post-SCI. The lack of SOD and MDA measurements at later days of SCI makes it difficult to conclude that recovery of oxygen free radical homeostasis precedes the neuromotor function recovery after the HBO treatment. The role of HBO treatment in the relationship between oxygen free radicals and acute SCI needs further study.

## Conclusions

In summary, our study shows a therapeutic effect of HBO treatment on SCI rats, demonstrated by improved hind limb neuromotor function. Enhanced serum SOD activity and decreased serum MDA content both indicate a potential role of oxygen free radicals in the pathophysiological consequence of SCI and its attenuation by HBO therapy.

## Abbreviations

BBB: Basso, Beattie, and Bresnahan locomotor rating scale
HBO: Hyperbaric oxygen
MDA: Malondialdehyde
SCI: Spinal cord injury
SOD: Superoxide dismutase

## References

[1] Hall ED (1993). Lipid antioxidants in actual central nervous system injury. Ann Emerg Med.

[2] Hall ED (1991). Inhibition of lipid peroxidation in CNS trauma. J Neurotrauma.

[3] Dayan K, Keser A, Konyalioglu S (2012). The effect of hyperbaric oxygen on neuroregeneration following acute thoracic spinal cord injury. Life Sci.

[4] Geng C-K, Cao H-H, Ying X (2015). The effects of hyperbaric oxygen on macrophage polarization after rat spinal cord injury. Brain res.

[5] Thompson CD, Zurko JC, Hanna BF (2013). The Therapeutic Role of Interleukin-10 after Spinal Cord Injury. J Neurotrauma.

[6] Yang Y, Piao Y (2002). Establishmentand evaluationof a SCImodel. Chin J Traumatol.

[7] Xia L, Sun Y, Sun S, Li J, Niu Y. The effect of PPAR-β on spinal cord and hind limb motor function in rats with spinal cord injury. Zhejiang Clinical Med. 2014;11:1717–9.

[8] Fehlings MG, Tator CH (1995). The relationship among the severity of spinal cord injury, residual neurological function, axon counts and counts of retrogradely labelled neurons after experimental spinal cord injury. ExpNeurol.

[9] Basso DM, Beattie MS, Bresnahan JC (1996). Graded histological and locomotor outcomes after spinal cord contusion using the NYU weight-drop device versus transection. ExpNeurol.

[10] New P (2008). Inappropriate suggestion of benefit from hyperbaric oxygen for spinal cord injury. Spinal Cord.

[11] Yin D, Zhou C, Ku S (2003). Et a1. Inhibition of apoptosis by hyperbaric oxygen in rat cerebral ischemic model. Cereb Blood Flow Metab.

[12] Lu P, Feng H, Wang X (2008). Effect ofhyperbaricoxygen preconditioningon neuron apoptosisafter acutespinal cord injury. Chin J Minim Invasive Neurosurg.

[13] Yaman O, Yaman B, Aydın F (2014). Hyperbaric oxygen treatment in the experimental spinal cord injury model. Spine J.

[14] Liu X, Zhou Y, Wang Z (2014). Effect of VEGF and CX43 on the promotion of neurological recovery by hyperbaric oxygen treatment in spinal cord-injured rats. Spine J.

[15] Sun LM (2008). Marti, Hugo H, et a1.Hyperbaric oxygen reduces tissue hypoxia and hypoxia-inducible factor-1 expression in focal cerebral ischemia. Stroke.

[16] Yu YM, Yukihiro M, Makoto Y (2004). Effects of hyperbaric oxygen on GDNF expression and apoptosis in spinal cord injury. Neuro report.

[17] Fang J, Hou T, Fang Y (2002). Effect of hyperbaric oxygen on excitatory amino acids in rat spinal cord injury. PLA Journal of Medicine.

[18] Hall ED, Braughler JM (1986). Role of lipid peroxidation in post traumatic spinal cord degeneration:a review in CNS trauma. Cent Nerv Syst Trauma.

[19] Sun Y, Liu D, Su P, Lin F, Tang Q. Changes in autophagy in rats after spinal cord injury and the effect of hyperbaric oxygen on autophagy. Neurosci Lett. 2016 3;618:139–145. 10.1016/j.neulet.2016.02.054 26949182

